# Structural and Functional Analysis of Excised Skins and Human Reconstructed Epidermis with Confocal Raman Spectroscopy and in Microfluidic Diffusion Chambers

**DOI:** 10.3390/pharmaceutics14081689

**Published:** 2022-08-13

**Authors:** Dorottya Kocsis, Hichem Kichou, Katalin Döme, Zsófia Varga-Medveczky, Zsolt Révész, Istvan Antal, Franciska Erdő

**Affiliations:** 1 Faculty of Information Technology and Bionics, Pázmány Péter Catholic University, H-1083 Budapest, Hungary; 2 EA 6295 Nanomédicaments et NanoSondes (NMNS) Laboratory, University of Tours, 37000 Tours, France; 3 Department of Pharmaceutics, Semmelweis University, H-1092 Budapest, Hungary; 4 Révész Plasztika, H-1125 Budapest, Hungary

**Keywords:** skin barrier, transdermal delivery, skin penetration, confocal Raman spectroscopy, skin-on-a-chip microfluidic device, hydration, transepidermal water loss, human-reconstructed epidermis

## Abstract

Several ex vivo and in vitro skin models are available in the toolbox of dermatological and cosmetic research. Some of them are widely used in drug penetration testing. The excised skins show higher variability, while the in vitro skins provide more reproducible data. The aim of the current study was to compare the chemical composition of different skin models (excised rat skin, excised human skin and human-reconstructed epidermis) by measurement of ceramides, cholesterol, lactate, urea, protein and water at different depths of the tissues. The second goal was to compile a testing system, which includes a skin-on-a-chip diffusion setup and a confocal Raman spectroscopy for testing drug diffusion across the skin barrier and accumulation in the tissue models. A hydrophilic drug caffeine and the P-glycoprotein substrate quinidine were used in the study as topical cream formulations. The results indicate that although the transdermal diffusion of quinidine is lower, the skin accumulation was comparable for the two drugs. The various skin models showed different chemical compositions. The human skin was abundant in ceramides and cholesterol, while the reconstructed skin contained less water and more urea and protein. Based on these results, it can be concluded that skin-on-a-chip and confocal Raman microspectroscopy are suitable for testing drug penetration and distribution at different skin layers within an exposition window. Furthermore, obese human skin should be treated with caution for skin absorption testing due to its unbalanced composition.

## 1. Introduction

During the last decad, an increasing interest can be observed in the field of use of in vitro skin substituents for the reduction in the number of experimental animals sacrificed for pharmaceutical and dermatological studies. The most relevant models are, of course, human skin, but the availability of these tissues is very limited and costly. The main advantage of the in vitro reconstructed skin equivalents is that they can be generated from human cells, and therefore, they are relevant, have good predictive potency and a low variability. The use of full-thickness three-dimensional (3D) skin models and human-reconstructed epidermis (HRE) is widespread in irritation, penetration and toxicological studies [1,2,3,4,5]. However, the structural mapping of these tissues and, in parallel, their functionality has not been analysed in detail yet.

The aim of the current experiments was to study the chemical composition of excised rat skins (with or without mechanical pretreatment), the EpiDerm^TM^ HRE (MatTek, Bratislava, Slovakia) and excised human skins from plastic surgery by confocal Raman spectroscopy and also to investigate their permeability and penetration capacity for two model drugs. The effect of the diffusion study itself on the ex vivo and in vitro skins (barrier function and epidermis composition) was evaluated at the end of skin-on-a-chip experiments as well. Caffeine was used as a hydrophilic model drug (logP = −0.07, [6]) and quinidine is a well-known P-glycoprotein (P-gp) substrate [7,8] with low water solubility (logP = 3.44, [9]). P-glycoprotein was found to be expressed in different cell types of the skin (keratinocytes, melanocytes, endothelial cells) [10,11,12,13], and its absorptive orientation has been reported by different research groups [14,15]. The skin-on-a-chip device is an appropriate platform to study the kinetics of transdermal drug delivery of different topical formulations in a dynamic diffusion system. Practically, it is a miniaturised version of a Franz diffusion cell with a direct connection between the tissue and the flowing perfusion media.

Based on the data in the literature, a three-step session has been developed. 

(1) First, a composition analysis was performed in the intact skin models. Six different characteristic components of the skins were analysed by Raman Spectroscopy. Ceramides are a family of waxy lipids, which are composed of sphingosine and a fatty acid. Ceramide is the main component of the *Stratum corneum* of the epidermis in human skin [16,17]. Ceramides, cholesterol and saturated fatty acids create a water-impermeable, protective structure to prevent water loss through the skin due to evaporation, and they make a barrier against the entry of microorganisms and external chemicals [17]. The *Stratum corneum* is composed of 50% ceramides, 25% cholesterol, and 15% free fatty acids [18]. Cholesterol synthesis is mainly essential for skin barrier homeostasis [19,20,21], but recent research has speculated that the *Stratum corneum* cholesterol domains may have a more complex role in the skin, other than a barrier limiting water loss and the entry of chemicals [22]. Numerous studies have shown that skin cholesterol content is associated with the deposition of cholesterol in the coronary arteries and aorta [23,24,25].

Lactate is a component of the natural moisturizing factor (NMF) and has a complex role in the skin. It stabilizes the pH, has a moisturizing effect (together with proteins, urea, lactic acid, sugar and inorganic ions such as potassium and magnesium ions), and provides anti-ageing and keratolytic action [26]. Due to these functions, sodium lactate is a frequently used ingredient in various cosmetics. Proteins and urea also belong to the NMF family and have moisturizing, rehydrating and anti-ageing effects. They are used both in dermatological products and in cosmetics.

The water content, e.g., the hydration, is a key aspect of the skin that influences its physical and mechanical properties. Hydration leads to changes in the molecular arrangement of the peptides in the keratin filaments as well as dynamics of C-H bond reorientation of amino acids in the protruding terminals of keratin protein within the *Stratum corneum*. The changes in molecular structure and dynamics occur at a threshold hydration (ca. 85% relative humidity) [27].

**Figure 1:**
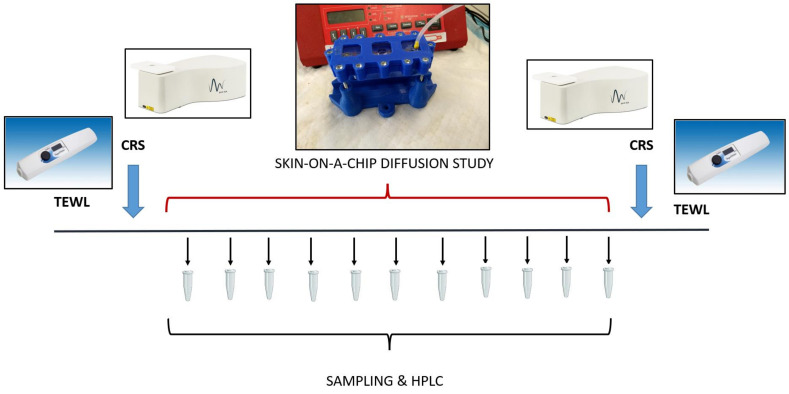
The experimental design for analysis of skin barrier function, skin composition and transdermal penetration of topical model drugs. TEWL: Transepidermal Water Loss; CRS: Confocal Raman Spectroscopy; HPLC: High-Performance Liquid Chromatography. The barrier function was measured at the beginning and at the end of the experiments on the skin and skin substitutes. Then the skin composition was analyzed by CRS, and this was followed by a skin-on-a chip diffusion study. The microfluidic samples were collected for 5 h every 30 min. Then, the accumulation of the test drugs was tested in the skin again by CRS.

(2) Then, the subjects were mounted in a skin-on-a-chip diffusion chamber and exposed topically to the cream formulations, and thereafter, a 5-h diffusion study was conducted.

(3) Finally, the tissues were analyzed again by confocal Raman spectroscopy, and the skin accumulation of the model drugs was determined in the epidermis. The transepidermal water loss (TEWL) was also measured before and after the diffusion experiments. The TEWL is an important parameter that is representative of the skin’s barrier function in vivo. In ex vivo experiments, it can serve as an indicator of the integrity of the skin barrier. The timeline of the experimental design is demonstrated graphically in Figure 1.

## 2. Materials and Methods

### 2.1. Solutions

In the skin-on-a-chip microfluidic device, peripheral perfusion fluid (PPF) was used as an extracellular fluid substitute acceptor solution in excised skin studies. It is composed of the following components: 147 mM NaCl, 4 mM KCl, and 2.3 mM CaCl_2_·2 H_2_O. All substances were acquired from Sigma-Hungary Kft, Budapest, Hungary. For human-reconstructed skin substitutes (HRE), Dulbeco-Phosphate-Buffered Saline (D-PBS) was used as a perfusion fluid provided by MatTek Life Sciences (Bratislava, Slovakia) in the EpiDerm^TM^ (Epi-212) kit.

### 2.2. Model Drugs and Formulations

Both model drugs (caffeine and quinidine) were purchased from Sigma Hungary Kft, Budapest, Hungary, and applied as a 2% suspension cream. In total, 2 g of caffeine/quinidine were dispersed with 4.1 g of liquid paraffin in a mortar and pestle. Then 47 g of white soft paraffin cream (containing polysorbate60 4%, white soft paraffin 26%, liquid paraffin 8 m/m%, propylene glycol 10%, cetostearyl alcohol 12 m/m%, and purified water 40%), 10 g propylene glycol, and 36.9 g of 0.21% citric acid aqueous solution were added.

### 2.3. Excised Skin Preparation 

Male Wistar rats (ToxiCoop, Budapest, Hungary) with 250–350 g bodyweight (2.5–3.5 months old) were used for skin preparation. The animals had free access to food and water before the study. The experiments were performed in compliance with the guidelines of the Association for Assessment and Accreditation of Laboratory Animal Care International and were in accordance with the spirit of the license issued by the Directorate for the Safety of the Food Chain and Animal Health, Budapest and Pest Agricultural Administrative Authority, Hungary (PE/EA/4122-7/2016). Rat skins were used for the skin composition and skin diffusion experiments. The animals were kept for at least 5 days in an animal facility (22 ± 3 °C, 50 ± 20% humidity and 12 h light-dark cycle) before the excision of the skin. Then, the rats were deeply anaesthetized with 400 mg/kg intraperitoneal (i.p.) chlorale-hyrate. The abdominal skin was shaved and epilated with a commercially available epilatory cream (ISANA^®^ cream from Rossmann, Burgwedel, Germany). After 5 min expostion time, the cream was removed by a wipe, and the skin surface was gently washed with tap water. After drying, the rat skins were either tape stripped 10 times (10TS) or left intact (0TS controls). The proper size of the tissue was then excised, and the samples were placed in a −80 °C deep freezer until the diffusion experiments. 

Human abdominal skin tissues obtained from plastic surgery clinics (Révész Plasztika, Budapest, Hungary) (permission number: 6501-6/2019/EKU, Budapest, Hungary) were used only for skin composition analysis experiments. The skins were collected from 42- and 44-year-old white female donors. The subcutaneous adipose tissue was gently dissected, and the rest of the skins were used in the skin composition experiments. The tissues were kept at −80°C until the day of the exposition or Raman experiments, and the tissues were taken from the freezer and transported at 4–8 °C in a cold room where they were defrosted overnight. The next day, the tissues were subjected to Raman analysis in the RiverD (GEN-2, Rotterdam, The Netherlands) system and then the rat skins were mounted in the skin-on-a-chip device, where the subcutaneous surface faced the peripheral perfusion fluid (PPF), and the *Stratum corneum* was exposed to a topical cream. The composition of PPF is described at Section 2.1.

### 2.4. Human Reconstructed Epidermis (HRE) Preparation

**Figure 2:**
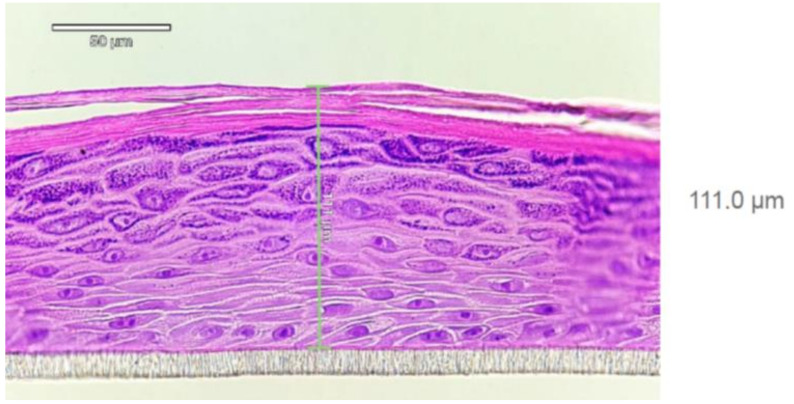
Histology report on EpiDerm tissue (HRE, EPI-200; LotNo:36125)) after hematoxilin-eosin staining provided by MatTek Life Sciences. Well-differentiated epidermis consisting of basal, spinous, granular layer of keratinocytes and *Stratum corneum* can be seen. At least 4 viable cell layers are present. The tissue thickness is 70–130 µm (average 111.0 µm).

EpiDerm^TM^ human-reconstructed in vitro epidermal tissues (0.6 cm^2^ of each) were purchased from MatTek Life Sciences, Bratislava, Slovakia. The histological structure of the HRE is shown in Figure 2. The 3D skin models were used within three days after delivery, and in the meantime, the tissues were kept at 4–8 °C in a cold room. The afternoon, before the day of the diffusion experiment, the tissues together with their transwell inserts were transported into a six-well plate (provided by the manufacturer) and placed into 0.9 mL medium (EPI-100-ASY assay medium, provided by the manufacturer) at 37 °C. The tissues were then incubated overnight (37 °C, 5% CO_2_). The next morning after 30 min hydration in Dulbeco-Phosphate-Buffered Saline (D-PBS) (TC-PBS-125, provided by MatTek Life Sciences, Bratislava, Slovakia) at 32 °C, the inserts were placed into the skin-on-a-chip device, and the perfusion was started at 4 µL/min flow rate, at 32 °C with D-PBS. This solution (D-PBS) was used as a receptor liquid. The test formulation (700–800 mg) was pipetted on the surface of *Stratum corneum* using MicroMan gel pipette (Gilson, Middleton, WI, USA), and the sample collection started immediately after. Thereafter, the perfusate samples were taken every 30 min for 5 h.

### 2.5. Skin-on-a-Chip Diffusion Studies

Similarly to the traditional Franz-diffusion cell system, the polydimethylsiloxane-(PDMS) based microfluidic chip is also composed of three functional elements: on top was a donor compartment, where the examined formulation was placed, the bottom was the receptor compartment, and in middle, an integrated skin sample (or a cell culture containing transwell insert) was placed, as described in details in our previous papers [15,28,29]. For excised skins, the so-called first-generation diffusion chambers were used, while for HRE, the second-generation devices were developed.

The diffusion surface of the skin was 0.50 cm^2^, and it was treated with 0.7–0.8 g of the different formulations (caffeine or quinidine creams, 2% of each) by a positive displacement piston gel pipette.

**Figure 3:**
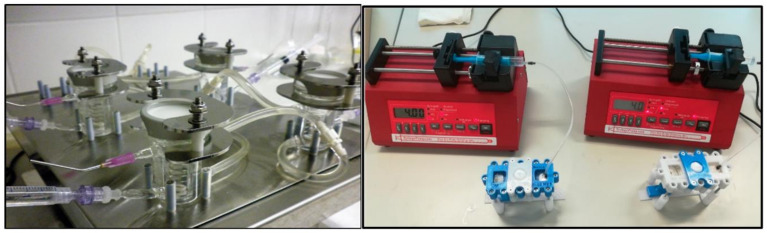
The static Franz diffusion cells (**left**) and the dynamic skin-on-a-chips (**right**). Both in the Franz cell and the skin-on-a-chip, the direction of the diffusion is vertical, but while in the Franz cell there is continuous stirring with a helical stirrer, in the chip, there is a continuous flow of the perfusion fluid below the skin sample.

Contrary to the static Franz-diffusion system, the microfluidic diffusion chamber is a dynamic system, where the flow is continuous below the treated skin surface in the subcutaneous area (Figure 3). The PPF or D-PBS solutions were loaded into a 5 mL syringe. Then a tube was connected to the syringe and the microfluidic chip, and the air bubbles were removed from the whole system, including the connected Teflon tubing and the microchannel of the chip. The flow rate was kept at 4 μL/min during the experiments, generated by a programmable syringe pump (NE-1000, New Era, Farmingdale, NY, USA). The whole setup was placed into a dry thermostat incubator at 32 °C for 6 h. The PPF/D-PBS solution was run through the chip, filling the receptor chamber reservoir and the microfluidic channel and leaving the device at the outlet into the collection vials. The perfusates were analysed by high-pressure liquid chromatography (HPLC) for caffeine or quinidine content immediately after the diffusion experiment.

### 2.6. Determination of Caffeine and Quinidine in the Perfusion Samples (HPLC)

High-performance liquid chromatography (HPLC) analysis was performed using an Ultimate 3000 (Thermo Fisher Scientific, Voisins-le-Bretonneux, France) piloted with Chromeleon 7.1 software (Thermo Fisher Scientific, Voisins-le-Bretonneux, France). A C18 column with a particle size of 5 μm and dimensions of 4.6 × 150 mm (Interchim, Montluçon, France) was used and adjusted on 25 °C. The software used was Chromeleon 7.1 (Thermo Scientific, chromatography data system software). 

Caffeine quantification: Samples were analyzed to quantify the concentration of caffeine and establish the time-dependent permeation kinetics using a DAD UV ultimate 3000 detector. Detection wavelength for caffeine was 272 nm. The mobile phase used in isocratic mode was methanol 50%: water 50% (*v*/*v*) + 10 mM of phosphoric acid. The injection volume of samples was 10 µL. The duration of the analysis of each sample was 6 min at a flow rate of 1 mL min^−1^. A standard calibration curve was prepared prior to analysis. Standards were obtained in triplicates by dissolving caffeine in PBS at concentrations of 1, 5, 10, 100 and 500 µg mL^−1^. The observed retention time was 2.621 ± 0.008 min. 

Quinidine quantification: Samples were analyzed using a DAD UV Ultimate 3000 detector. Absorbance was recorded at 250 nm. The analysis was performed in isocratic mode using an acetonitrile/water (10%/90%) mobile phase supplemented with 3 g of hexylamine. The pH of the mobile phase was adjusted to 2.8 by adding phosphoric acid, according to the European pharmacopoeia method. The injection volume of samples was 10 µL. The duration of the analysis of each sample was 5 min at a flow rate of 1 mL min^−1^. A standard calibration curve was prepared prior to analysis. Standards were obtained in triplicates by dissolving quinidine in the mobile phase at concentrations of 0.1, 0.5, 1, 5 and 10 µg mL^−1^. The observed retention time was 3.033 ± 0.005 min.

### 2.7. Confocal RAMAN Spectroscopy

Confocal Raman Spectroscopy analysis was performed with a GEN-2 SCA Skin Composition Analyser (River D—International BV, Rotterdam, The Netherlands) equipped with two incorporated lasers operating at different wavelengths. The device is regularly used for in vivo and ex vivo analysis of both human and porcine skin [30,31] and is able to monitor treatment-induced changes in skin parameters.

The first laser wavelength of 785 nm (25 mW on the skin) was used for the analysis of the skin fingerprint region (FP) (400–1800 cm^−1^). The second laser of 671 nm (20 mW on the skin) was used for the analysis of the high wave number (HWN) region (2500–4000 cm^−1^). 

For each skin sample, 10 areas were recorded using depth profiling (z-profiling) for both FP and HWN regions. The FP z-profilings were carried out with a 3 s exposition time and 4 µm steps up to 28 µm depth. For the HWN measurements, 1 s exposition time and 4 µm steps were used for z-profiling up 40 µm as a final depth. 

The instrument was calibrated daily using an integrated Neon-Argon lamp, which features peaks at precisely known emission wavelengths. The wavelength shift is calibrated using a polymethyl methacrylate (PlexiglasTM) standard. 

Skin components profiles, namely ceramides, fatty acids, cholesterol, lactate (pH = 4 was used because the spectrum of lactate used for the fitting model was found to be pH-dependent within the skin acidity range (pH 4.5–7), which varies from person to person, and the spectrum at pH = 4 was the best fitting with the real skin spectrum [32]), proteins, urea and water content were calculated using the SkinTools 3 analysis software (RiverD International B.V., version 3.3.201202, Rotterdam, The Netherlands) according to the previous work of Caspers et al. [32]. 

The quantification of caffeine and quinidine were calculated using the SkinTools 3 analysis software (RiverD International B.V., version 3.3.201202, Rotterdam, The Netherlands), according to the quantification method described by Caspers et al. [33]. 

### 2.8. Transepidermal Water Loss (TEWL)

Increased TEWL indicates skin barrier damage [34,35,36]. TEWL measurements were performed with the condenser-chamber device Vapometer^®^ (Delfin Technologies Ltd., Kuopio, Finland). With its closed-chamber principle, the Vapometer^®^ creates a microclimate within the measurement compartment. Thus, measurements remain unaffected by external air turbulences. The device measures water evaporating from the skin in g/m^2^/h. 

### 2.9. Data Analysis

Data analyses were performed using SkinTools 3 (RiverD International B.V., version 3.3.201202, Rotterdam, The Netherlands). The spectra were cut between 400 and 1800 cm^−1^ for the FP region. For the HWN region, the spectra were cut between 2500 and 3600 cm^−1^. A baseline subtraction was applied using a 3rd-degree polymer function.

TEWL values were compared before and after the diffusion experiments using paired Student t-test. *p* < 0.05 was confirmed as a statistically significant difference.

In the case of skin composition analysis, the Arbitrary Unit vs. Depths curves were calculated, and the individual dots were shown as means ± standard error of mean (SEM). The Area Under the Curve (AUC) values were generated from these plots by OriginPro 2015 Sr2 software (MacaSoft Bt, Győr, Hungary) with the trapezoid method. The AUC values were compared with human data using unpaired Student *t*-test. *p* < 0.05 was regarded as a statistically significant difference. In the case of HRE, only a few tissue samples (2 for composition studies, 1 for drug penetration experiments) were available; thus, these data can only be regarded as pilot results. Both on the excised skins and on the in vitro skin substituents, 8–10 different locations were analysed by Raman spectroscopy, and for HRE, the replicates showed very low variability.

## 3. Results

The composition of human skin, rat skin and the human-reconstructed epidermis was analysed by testing six different indicators: ceramides and fatty acids, cholesterol, lactate (pH = 4), water, urea and proteins. 

### 3.1. Composition Analysis of Excised Skins and In Vitro Epidermis (HRE) 

**Figure 4:**
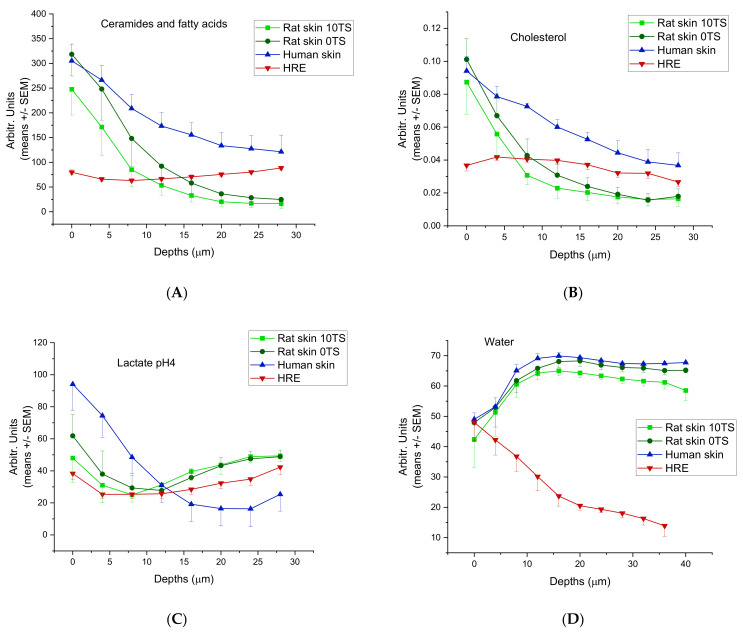

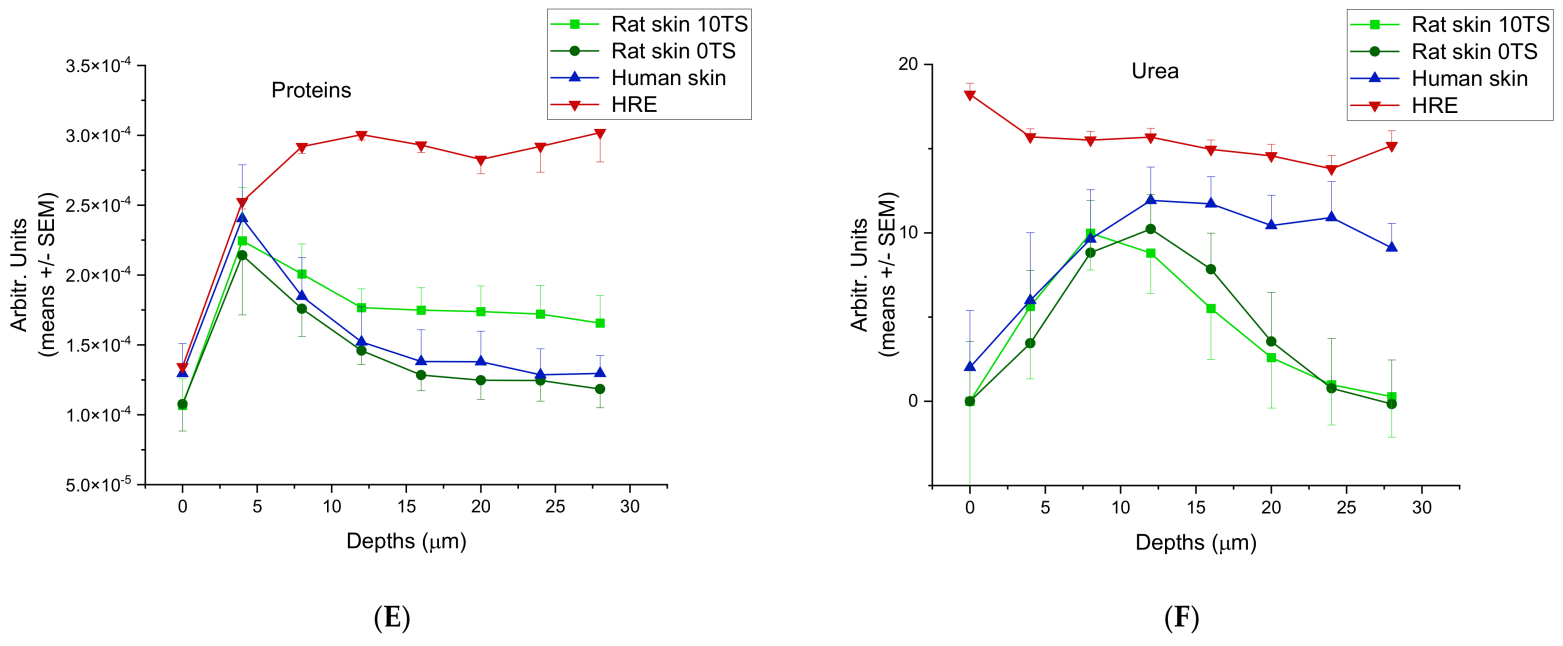
Skin composition profiling in 0–28 µm (for water 0–40 µm) depths from the outermost surface of *Stratum corneum* in intact (0TS) and mechanically pretreated (10TS) rat skins and intact human skin after freezing and thawing and also in in vitro skin substituents (HRE). (**A**) Ceramides and fatty acids, (**B**) cholesterol, (**C**) lactate (pH = 4), (**D**) water, (**E**) proteins, (**F**) urea. The experiments were performed in duplicates (in HRE) or in triplicates (in rat and human skins) using N = 8–10 different positions for z-profiling on all samples.

The ceramide/fatty acid composition of rat skin, HRE and human skin are presented in Figure 4A. The composition characteristics were similar in rat and human excised skins. (The highest values can be seen in the skin surface, and there is a monotonous decrease with the depths (from 300 to 110 Arbitr. Units in human, and from 300/intact 0TS/or 250/sensitised 10TS/to 20–25 Arbitr. Units in rats). These data indicate that the tape stripping process (mechanical sensitisation) results in loss of ceramide/fatty acid components at the surface of *Stratum corneum*. The HRE showed a very low ceramid/fatty acid level (75–100 Arbitr. Units), which was moderately increasing with the depth. Cholesterol (Figure 4B) was also similar in excised skins (human and rat) (0.09–0.1 Arbitr. Units on the surface and 0.04–0.02 Arbitr. Units at 28 µm depth), but different in EpiDerm^TM^ (HRE). In the reconstructed tissue, constantly low cholesterol content can be seen (0.04–0.03 Arbitr. Units) both on the surface and in the deeper layers. Lactate profile (Figure 4C) was comparable in rat skin and HRE, but different in human tissue, showing a higher lactate level in the outermost layer of the *Stratum corneum*, and decreasing values at deeper z-profiles. The water content (Figure 4D) peaked at about 12–16 µm depths at the border zone of dead and viable epidermis in rat and human skins, while in HRE, the highest hydration can be observed on the surface.

The proteins (Figure 4E) and urea (Figure 4F) showed similar characteristics in human and rat skins with a maximum concentration at 8 and 12 µm depths for urea and at 4 µm for proteins, respectively. On the contrary, HRE has the maximum urea content on the outermost surface of *Stratum corneum* and the protein levels were increasing to 8 µm depths and then showed a plateau level in the whole thickness studied (until 28 µm depths).

**Figure 5:**
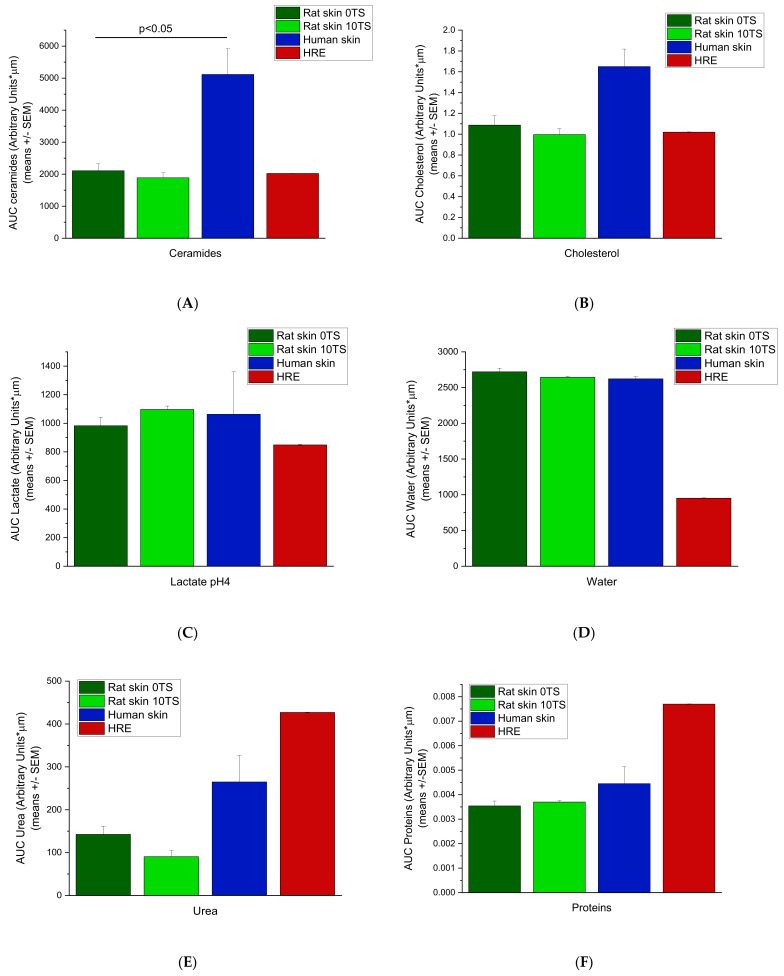
Area under the skin depths—Arbitrary Units curves (AUC) for intact rat skins (0TS), mechanically pretreated rat skins (10TS), intact human skins and EpiDerm^TM^ human-reconstructed epidermis (HRE). Measurements were taken on the epidermis at 8–10 different locations for 0–40 µm depths for water and for 0–28 µm depths for all the other skin components. (Number of subjects = 3–2, with N = 8–10 locations for each). (**A**) Ceramides, (**B**) Cholesterol, (**C**) Lactate pH = 4, (**D**) Water, (**E**) Urea, (**F**) Proteins.

To characterise the whole amounts of the components in the epidermis, AUC values were calculated (Figure 5A–F) for each compound (or compound family) using the Arbitrary Unit-Depths profiles. For the lipid components (ceramides, fatty acids and cholesterol) (Figure 5A,B), the highest amounts were determined in the excised human skins, which differed from the rat skins in a statistically significant manner (*p* < 0.05). The rat skin’s AUC values are very close to the HRE data (non-sensitised rat skin 2087 ± 215.97, sensitised rat skin 1886.5 ± 161.95 and HRE 2023 ± 3.98 Arbitr. Units*µm), but due to the low number of replicates in HRE, the difference from the human skin was not statistically significant. For lactate content (pH = 4) (Figure 5C), the excised human and rat skins showed similar data, while HRE showed lower levels (*p* > 0.05). The hydration of the ex vivo tissues was similar (non-sensitised rat skin 2720.3 ± 51.03; sensitised rat skin 2644.5 ± 14.15; human skin 2622.0 ± 31.02 Arbitr. Units* µm), while HRE showed much lower water content in the epidermis (952.2 ± 4.54 Arbitr. Units* µm) (Figure 5D). For urea (NMF component), the AUC value of HRE was the highest (Figure 5E), and also for protein (NMF component) content, the in vitro skin tissue (HRE) was the most abundant in proteins (Figure 5F), but the difference was not statistically significant due to the low number of replicates in artificial skin samples.

### 3.2. Transdermal Absorption of the Model Drugs (Caffeine and Quinidine)

**Figure 6:**
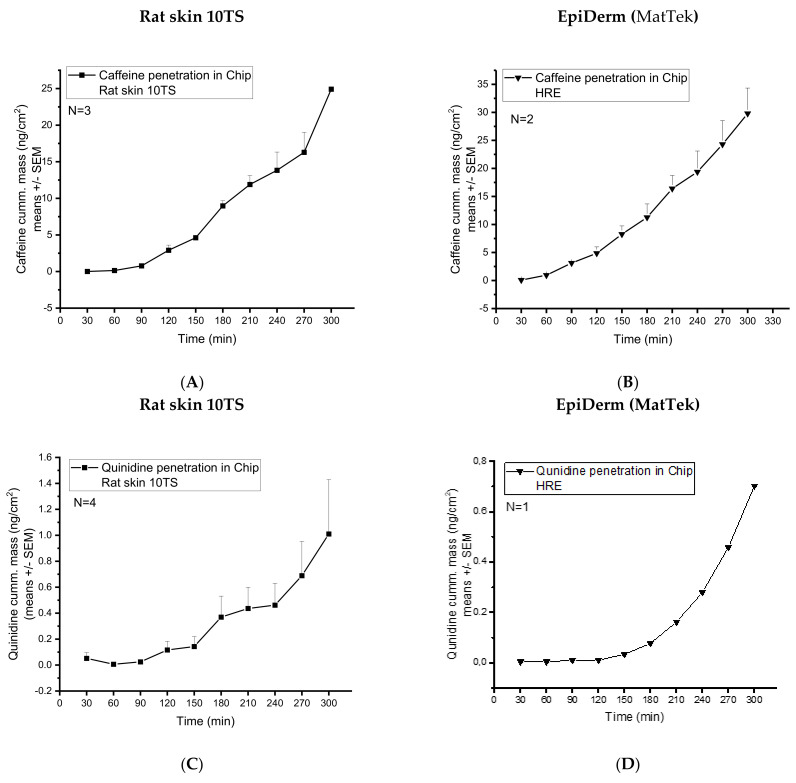
Transdermal diffusion (cumulative mass ng/cm^2^) of (1) caffeine in mechanically pretreated rat skins (**A**) and EpiDerm HRE tissues (MatTek) (**B**), and (2) quinidine in mechanically pretreated rat skins (**C**) and EpiDerm HRE tissues (MatTek) (**D**). The investigations were performed in a skin-on-a-chip microfluidic device. N = 3−4 for rat skins and N = 2−1 for EpiDerm human-reconstructed epidermis model.

Previous studies demonstrated that the caffeine and quinidine penetration through the non-sensitised rat skin is under the detection limit by our analytical techniques. Therefore, for the drug-penetration studies, only the 10TS, mechanically sensitised rat skins were used. Caffeine penetration from cream formulation through the rat skin (10TS) and HRE was investigated in skin-on-a-chip diffusion chamber. The concentration time-profiles of the two different diffusion models were similar (Figure 6A,B). The cumulative amounts reached 24.92 ± 0.25 and 29.79 ± 4.54 ng/cm^2^ values after 5 h dynamic perfusion with PPF/D-PBS (Figure 6A,B).

Quinidine penetration from cream formulation through the rat skin (10TS) and HRE was also investigated in the skin-on-chip diffusion chamber. The concentration time-profiles of the two different diffusion subjects were similar in shape, but the transepidermal diffusion was moderately higher in the excised skins than in the reconstructed tissue. The maximum cumulative amounts of quinidine reached were 0.7 ng/cm^2^ in HRE and 1 ng/cm^2^ in rat skins after 5 h dynamic perfusion with PPF/D-PBS (Figure 6C,D). 

### 3.3. Tissue Accumulation of the Model Drugs (Caffeine and Quinidine)

**Figure 7:**
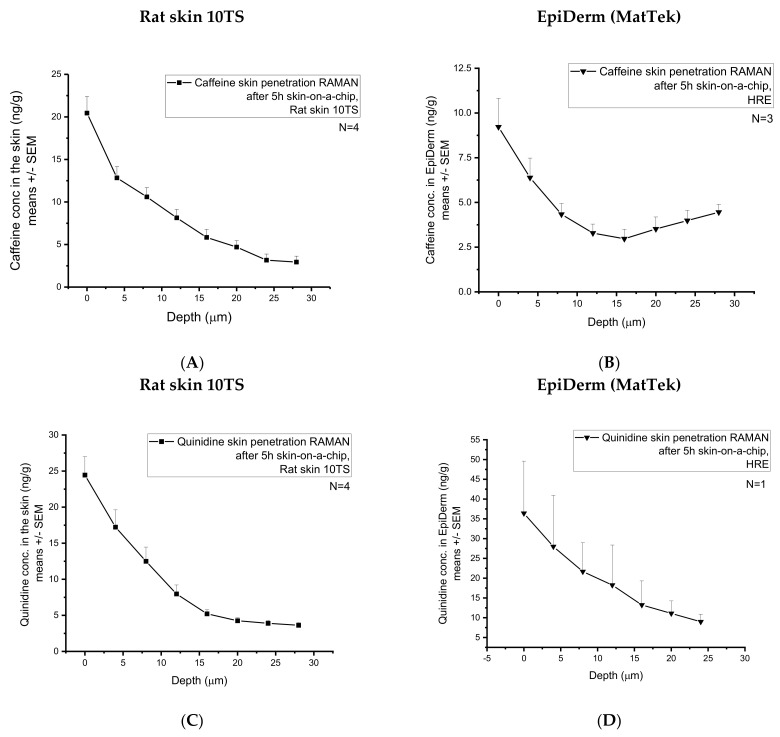
Accumulation of the two model drugs (caffeine and quinidine) in the skins and skin substitutes determined by Confocal Raman Spectroscopy. Caffeine penetration in mechanically pretreated rat skins (**A**) and in EpiDerm HRE tissues (MatTek) (**B**). Quinidine penetration in mechanically pretreated rat skins (**C**) and in EpiDerm HRE tissues (MatTek) (**D**). Four rat skins and 1-3 EpiDerm human-reconstructed epidermis were studied. N = 8–10 different z-profiling positions were applied for each individual sample.

After the skin-chip study, the accumulated caffeine content was also analyzed at eight different skin depths from 0 to 28 µm) in the subjects (rat skin 10TS and HRE) (Figure 7A,B). As it can be seen in the Raman spectra (Figure 7A,B), the full-thickness excised skin accumulated approximately twice more caffeine than HRE (from 20.44 ± 1.94 to 2.93 ± 0.70 ng/g vs. from 9.22 ± 1.59 to 4.5 ± 0.44 ng/g measured from the surface until the 28 µm depth). These results can be explained by the higher water content of the rat skin than the HRE, as it is shown in Figure 4D and Figure 5D.

A large standard deviation was observed, indicating a large degree of intra-sample variability. Indeed, skin is a complex biological matrix, and it can be anticipated that the distribution of caffeine/quinidine across the *stratum corneum* (28 µm) would display a level of heterogeneity, inherent in biological systems, which are heterogeneous by nature. The heterogeneous structure of the corneocytes embedded in the lipid matrix of the *stratum corneum* results in an uneven distribution of the hydrophilic caffeine.

After the skin-chip study, the accumulated quinidine content was also analysed at eight different skin depths from 0 from 28 µm in the two subjects (Figure 7C,D). As it can be seen in the Raman spectra, the full-thickness excised skin accumulated less quinidine than the HRE. As quinidine is a relatively lipophilic and non-water-soluble compound, the low hydration of the reconstructed epidermis might be a better chemical environment than the skin for the accumulation of this substance. 

Comparing caffeine and quinidine diffusion (Figure 6A,B vs. Figure 6C,D), the degree of absorption across the skin or skin substitutes is much higher in the case of caffeine, indicating that caffeine is more soluble both in the cream formulation and in the extracellular matrix of the skin than quinidine. In excised tissues, caffeine is able to penetrate across the barrier through the transappendageal route [37] and easily diffuse into the dermis and subcutis, which is not the case in HRE. In the in vitro tissues, there are no hair follicles, sweat and sebaceous glands and ducts that mediate the penetration. On the other hand, quinidine is the substrate of several efflux transporters (especially P-gp), which are expressed in various skin cells. Therefore, although the transepidermal (transappendageal) diffusion of quinidine is lower, the accumulation is similar or even higher in the skin (Figure 7A–D) than that of the caffeine, as indicated by Raman spectroscopy. The higher amount of quinidine in HRE than in rat skin (Figure 7C vs. Figure 7D) can be the consequence of the higher number of active P-gp efflux pumps in the in vitro skins because the excised rat skins were subjected to a freezing and thawing process, which leads to degradation of transporter proteins.

### 3.4. Epidermal Barrier Function

**Figure 8:**
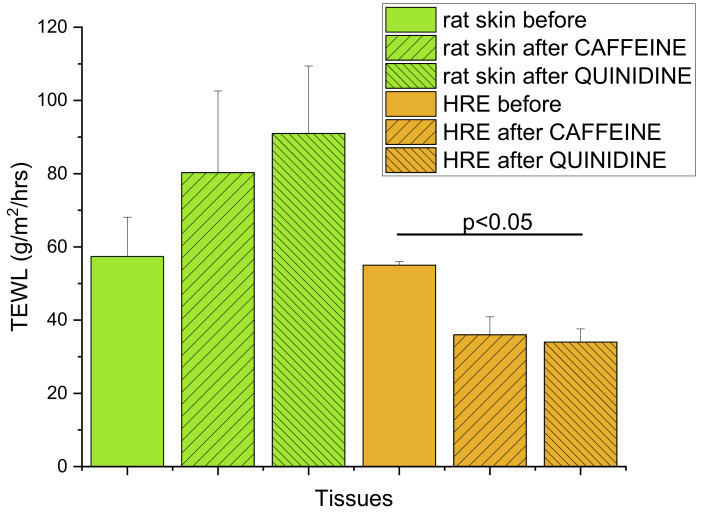
Transepidermal water loss (TEWL) as the indicator of dermal barrier function, determined in (1) rat skin samples after 10 tape strippings before any kind of treatment or after 5h exposition to 2% caffeine or quinidine cream in the skin-on-a-chip device; (2) in EpiDerm^TM^ HRE tissues before any kind of treatment or after 5 h exposition to 2% caffeine or quinidine cream in the skin-on-a-chip device. N = 11 for the skin before, and N = 3−4 for the other groups. Data are expressed as means ± SEM.

The tapered-off *Stratum corneum* (10TS) of excised rat skins showed a similar barrier function to HRE, as indicated by the TEWL values in Figure 8 (57.4 ± 10.7 and 56.0 ± 1.0 g/m^2^/h, respectively) before the diffusion experiments. However, after 5h exposition to drug-containing creams (caffeine or quinidine) and continuous perfusion in skin-chip, an increasing permeability was demonstrated in rat skins (80.3 ± 22.3 and 91.0 ± 18.4 g/m^2^/h), and lower TEWL could be seen in HRE (36.0 ± 4.9 and 34.0 ± 3.6 g/m^2^/h). The difference between the TEWL values of control HRE and HRE after quinidine exposure was statistically significant (*p* = 0.0356 by Student-*t* test). The increased TEWL in excised skins can be the consequence of the swelling of the tissue and changes in the hydration during the 5h perfusion. Impairment of barrier function was also described in relation to the lipid and protein properties of the *Stratum corneum* in human-reconstructed skin equivalents by other authors [38]. The standard error of mean values (SEM) and, therefore, the variability of the model was much lower in reconstructed epidermis than in rodent skins.

## 4. Discussion and Conclusions

The topical administration of drugs on the skin is challenging. Although this route of administration allows the treatment of both local and systemic pathologies, the skin is a strong barrier against xenobiotics. The outermost layer of the skin, namely the *Stratum corneum*, a multi-layered wall-like structure in which flat keratinised corneocytes are embedded in a lipophilic network, is difficult to cross by exogenous molecules. Thus, this route of administration is limited to a few compounds with defined physicochemical properties. However, several new physical, chemical and formulation technologies were developed during the last years [39,40,41,42] to improve drug penetration through the dermal barrier [43]. For testing chemical absorption in the skin, regulated methods are available. In this study, a new experimental tool (skin-on-a-chip) and one of the most advanced Raman spectroscopic techniques (confocal Raman spectroscopy) were combined for the purpose of comparison of natural and artificial tissues.

Reconstructed human epidermis (HRE) was used for analysing the transepidermal diffusion and accumulation of small molecule model drugs in skin-on-a-chip and by confocal Raman spectroscopy. The current setup is feasible for applying transwell inserts directly on the chips (second generation device). The dynamics, reserved by dermal microcirculation, was also considered using continuous slow perfusion of the device across the microfluidic channel with physiological solutions. Some more complex systems were recently reported, where the tissue flexibility, the capillary endothelial network and the immunological cell types (e.g., HL-60 leukocytes) are also included in the microfluidic system [44,45]. Future research should focus on the creation of more physiological in vitro systems with their own vascularity and immunological components to provide more appropriate structure and composition of the artificial tissue. The current investigations can be regarded as an early stage discovery research, which makes a relatively rapid screening of the test drugs for barrier penetration and tissue accumulation possible. As the main components of the skin barrier are localised in *Stratum corneum* and granular epidermis (tight junctions), HRE also includes the most important elements of this barrier.

The basic questions of the current study were (1) does the 5 h diffusion cell study have any effect on the properties of the skins/skin equivalents? (2) Can the differences be observed between the composition of human skin, rat skin and HRE by Raman analysis? (3) Can the transdermal diffusion be measured in parallel with the skin accumulation of the test drugs (caffeine and quinidine)? (4) Can the human skins be replaced with rat skins or with HRE in skin-on-a-chip diffusion experiments?

Based on the results, the following answers can be given to the above questions: (1) In preliminary experiments, the rat skins were tested in skin-on-a-chip device without topical treatment and with 5 h topical treatment with blank cream (data are not shown). No difference was observed in the skin composition before and after the study (Supplement Materials Figure S1). In the second series of the experiments, 5 h topical treatment with caffeine/quinidine creams was applied and no difference was found in ceramide, cholesterol, lactate, water, urea and protein contents (for spectra before and after the diffusion studies, see Supplement Materials Figure S2). The barrier function of the skins was also checked before and after the drug diffusion study, and impaired permeability was found in animal skin, but lower TEWL was seen in HRE. These results indicate that HRE is more resistant to the shear stress occurring in the skin-chip and also the placement of transwell inserts containing HRE to the chip is non-effective on the tissue (second generation device), contrary to the tension, which is applied on the border zone of skin tissues during fixation into the chip (first generation device). Furthermore, a high increase in hydration could be observed in rat skins after the diffusion experiments. (2) Remarkable differences in the skin composition can be detected in ceramide, fatty acids and cholesterol contents. The level of these factors is much higher in the human skin tissues in our experiments, which can be explained by the obesity of the donors, but further studies on healthy human skin are proposed. This observation indicates that not all human skin samples can be good for pharmaceutical or dermatological studies. Sometimes the donors from plastic surgery are not well-comparable with the healthy individuals, and a normal animal skin or HRE can be more relevant or more predictive of the physiological conditions. The next difference between the subjects tested is that the HRE contains less water but more elements of the natural moisturising factor (NMF), such as urea or proteins, than the natural skin tissues. (3) This study provided evidence that the three-step study protocol is feasible for studying skin composition-penetration and accumulation in skins and skin equivalents in topical drug developments. (4) As it is shown in Figure 6, the diffusion study provided comparable results in rat skin and in HRE, both in the case of caffeine and quinidine. Furthermore, the data are more even, and the variability is much lower in the artificial tissues. However, it is important to mention that these experiments in human skin equivalents were performed only on a low number of subjects. To make a statistical confirmation of the results, further experiments are needed.

In summary, the findings of current research confirm that HRE can be a good substitute for human or animal tissues in diffusion studies, also in skin-on-a-chip systems, although there are some differences in the composition of the structural elements between HRE and skins. The pathological human obese skin tissues should be compared with healthy tissues in future studies to explore whether the obese skins are relevant models in pharmaceutical studies or if HRE is preferred. For prediction of drug penetration and accumulation in physiological or dermatological conditions (such as psoriasis, atopic dermatitis and inflamed or dry scaly skin), further Raman analysis and diffusion studies are proposed in ex vivo tissues from preclinical disease models [46,47,48]. Furthermore, to analyse the skin composition in the various dermatological conditions, further studies are needed.

## Data Availability

The data presented in this study are available on request from the corresponding author.

## Supplementary Materials

The following supporting information can be downloaded at: https://www.mdpi.com/article/10.3390/pharmaceutics14081689/s1, Figure S1: Raman spectra of rat skins before and after skin-on-a-chip with 5 h blank cream exposure. Figure S2: Raman spectra of rat skins before and after skin-on-a-chip and 5 h caffeine (**A**,**B**) or quinidine (**C**,**D**) exposure.
