# Supplementary material for: Structural and Functional Analysis of Excised Skins and Human Reconstructed Epidermis with Confocal Raman Spectroscopy and in Microfluidic Diffusion Chambers
Source: Pharmaceutics. 2022 Aug 13;14(8):1689. doi: 10.3390/pharmaceutics14081689 (PMC9415586; doi:10.3390/pharmaceutics14081689)
Supplement: Supplementary file 1 [file pharmaceutics-14-01689-s001.zip › pharmaceutics-1840450-supplementary.pdf]

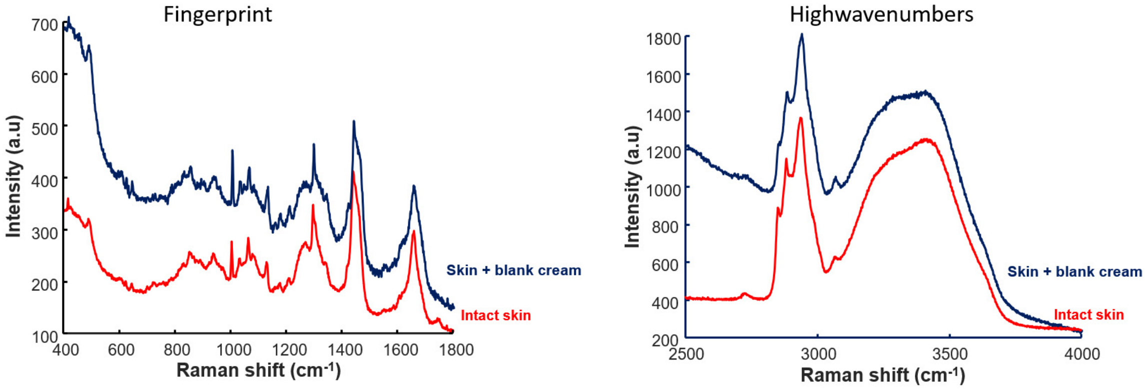

Figure S1. Raman spectra of rat skins before and after skin-on-a-chip with 5h blank cream exposure. The figure shows the mean spectra of 0 µm to 28 µm depths for FP and of 0 to 40 µm for HWN region, n = 40. The spectra demonstrate the main characteristic features observed originated from the lipids, nucleic acids, and proteins which are found in the skin (1655, 1440 and 1469 , 1303, and1005 cm^{-1}) and are present at the same position (no shift) before and after the skin-on-the-chip experiment. These results indicate that chip experiment using blank cream do not impact the skin composition.

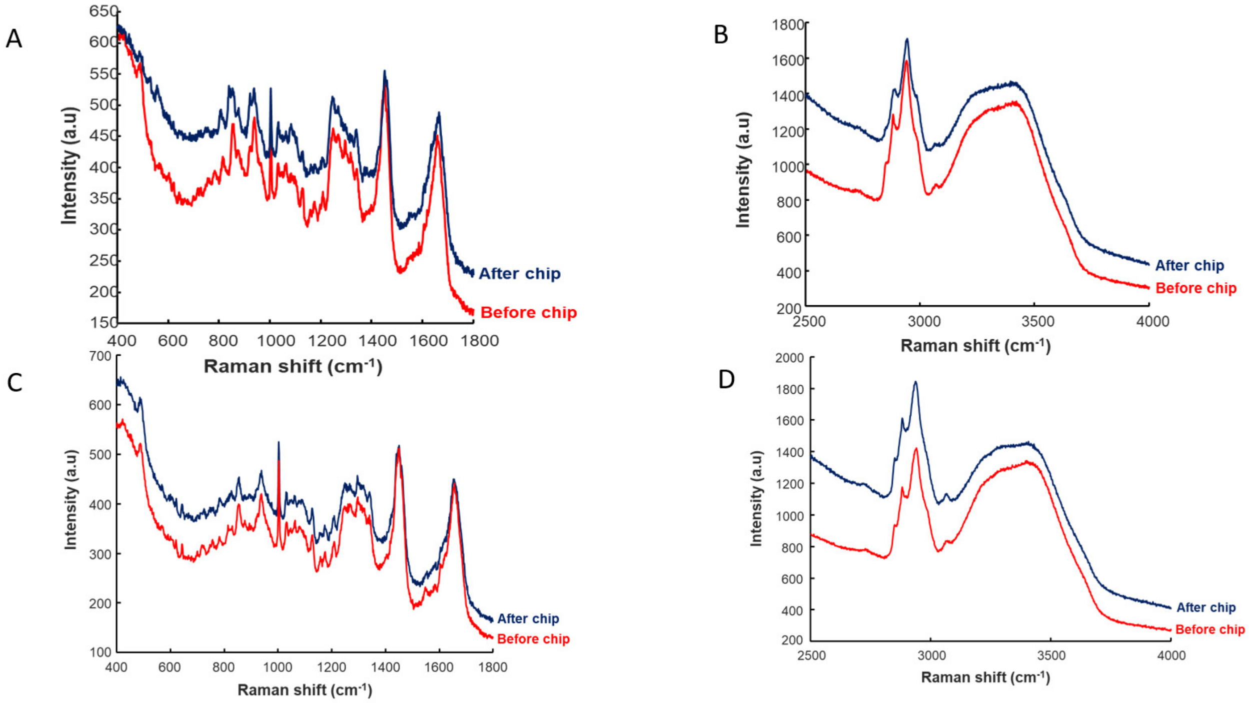

Figure S2. Raman spectra of rat skins before and after skin-on-a-chip and 5h caffeine (**A**,**B**) or quinidine (**C**,**D**) exposure. The figure shows the mean spectra of 0 µm to 28 µm depths for FP and of 0 to 40 µm for HWN region, n= 40. The spectra demonstrate the main characteristic features observed originated from the lipids, nucleic acids, and proteins which are found in the skin (1655, 1440 and 1469 , 1303, and1005 cm^{-1}) and are present at the same position (no shift) before and after the skin-on-the-chip experiment. These results indicate that chip experiment using caffeine or quinidine creams do not impact the skin composition.
